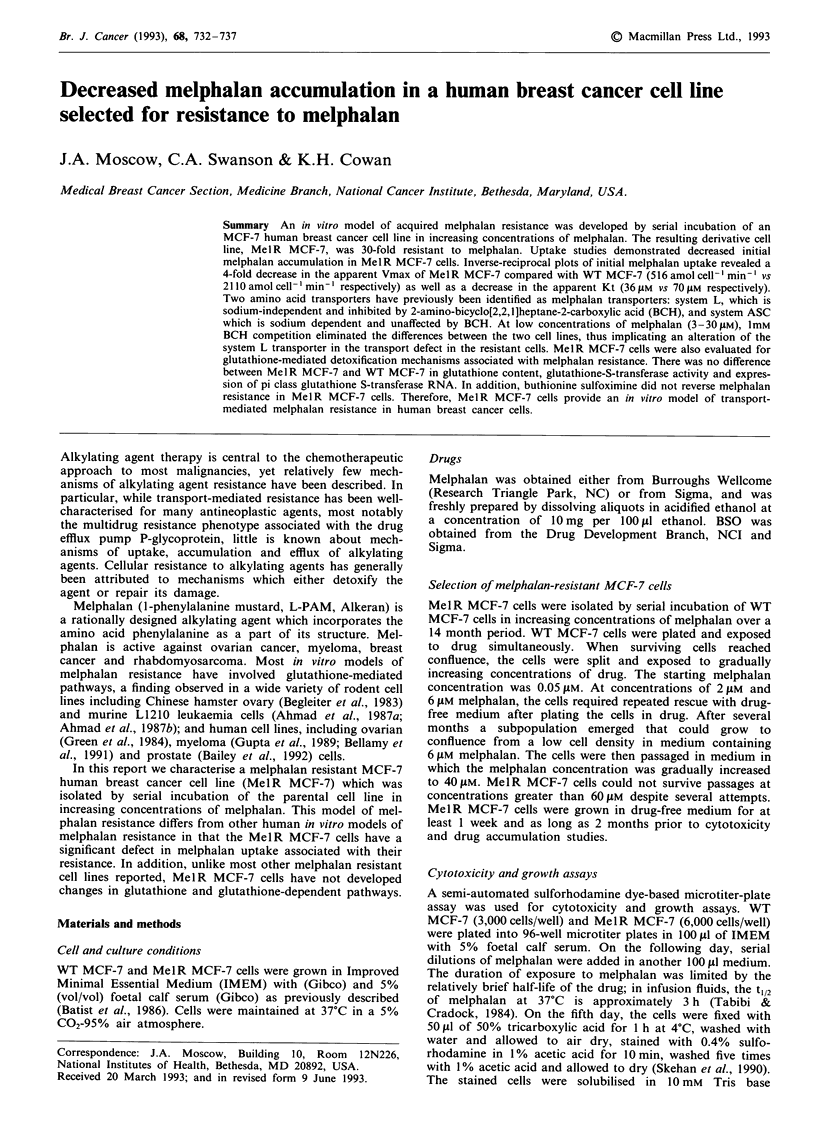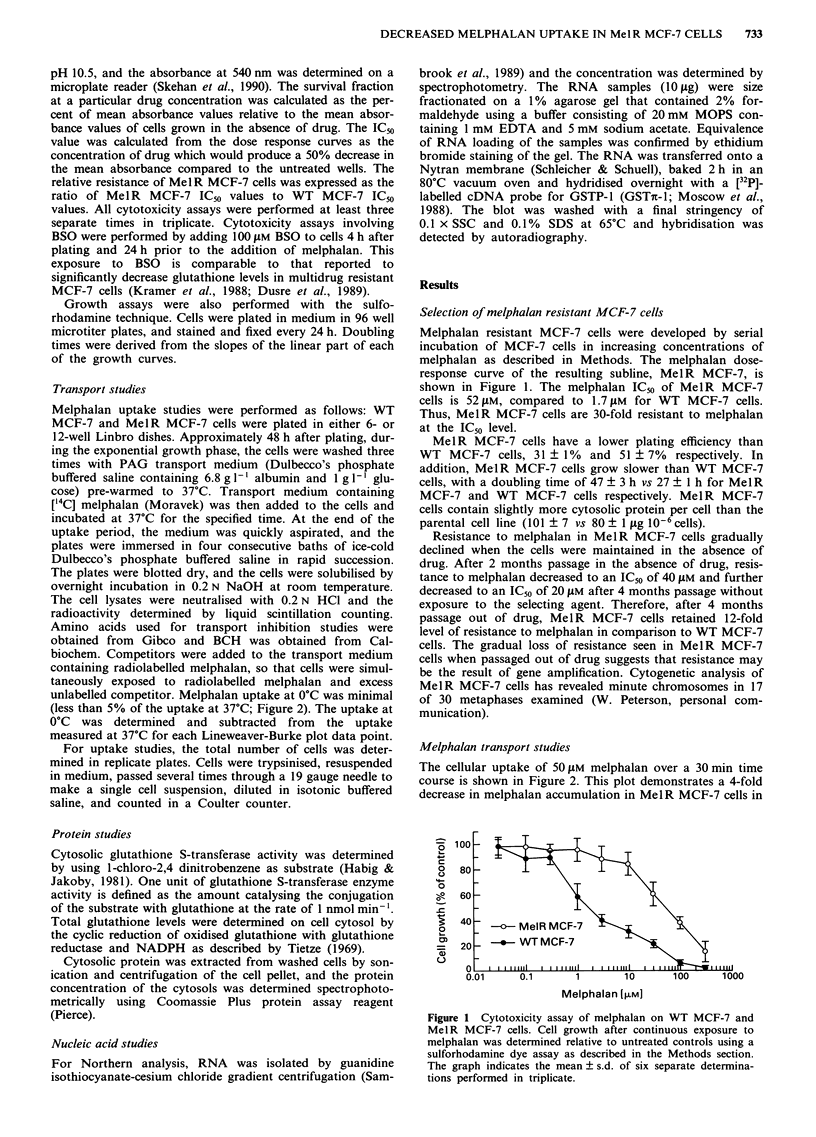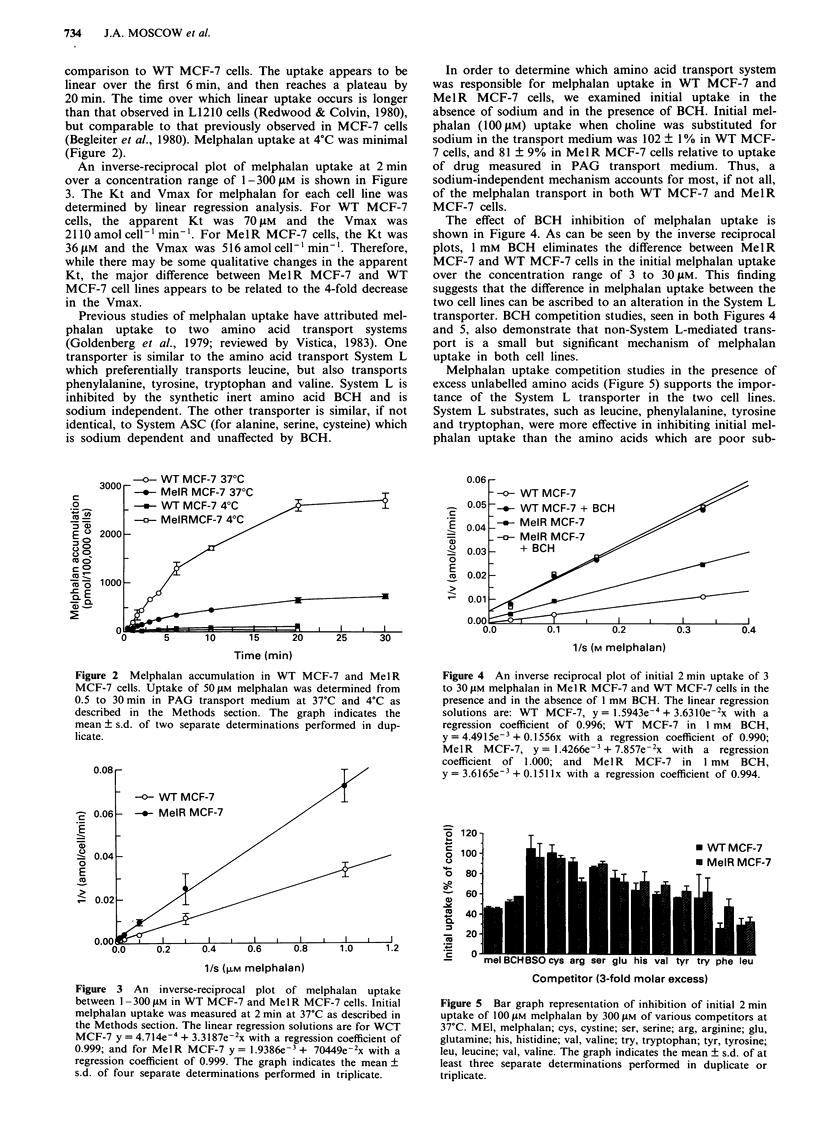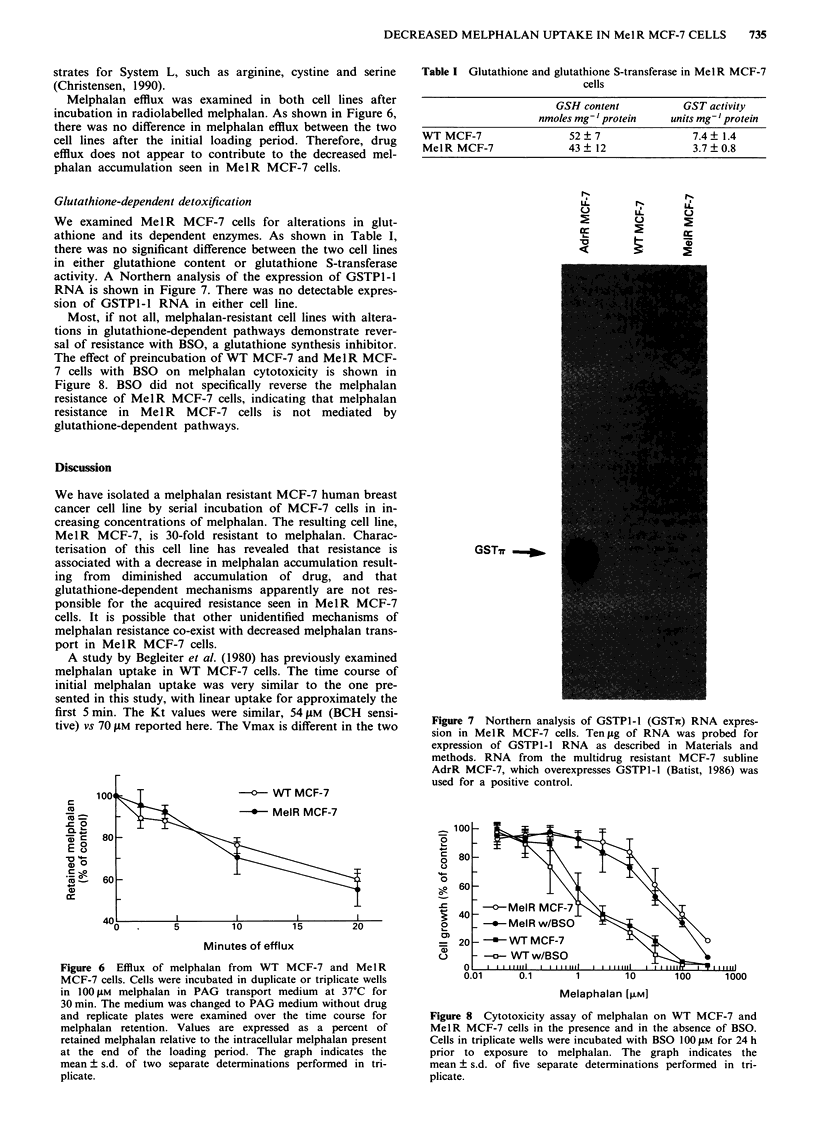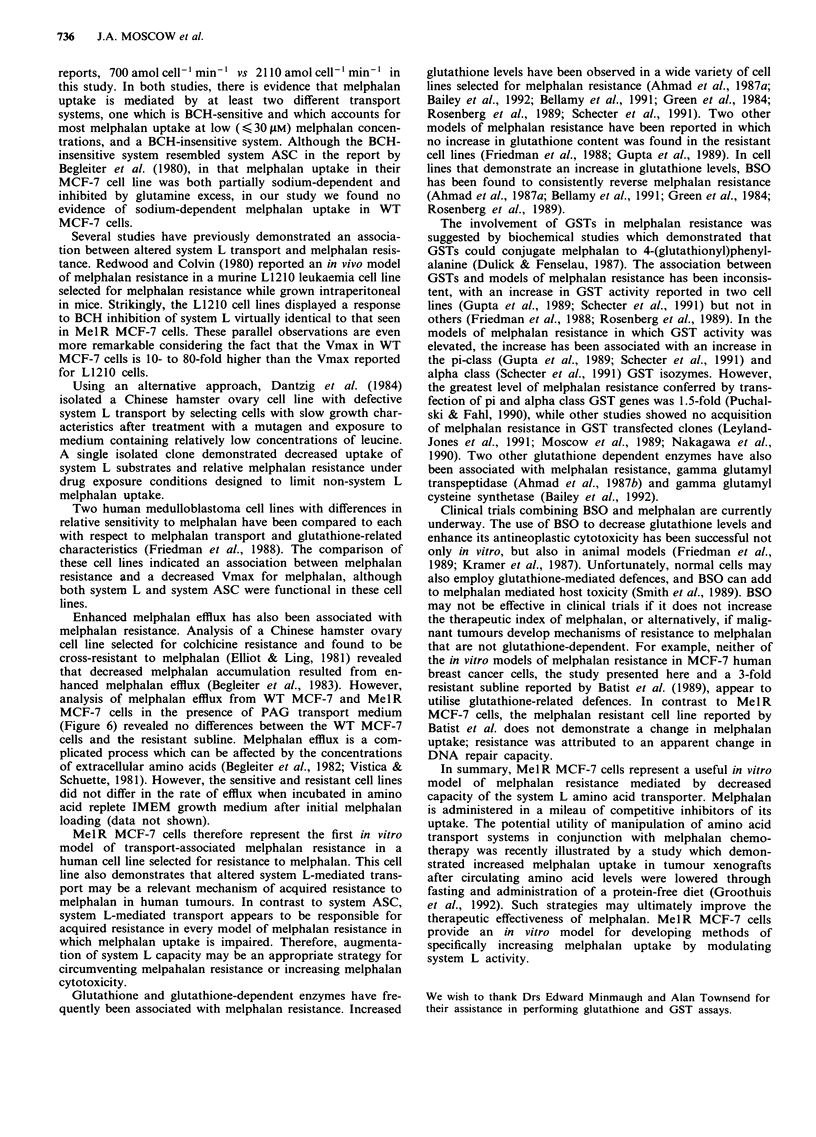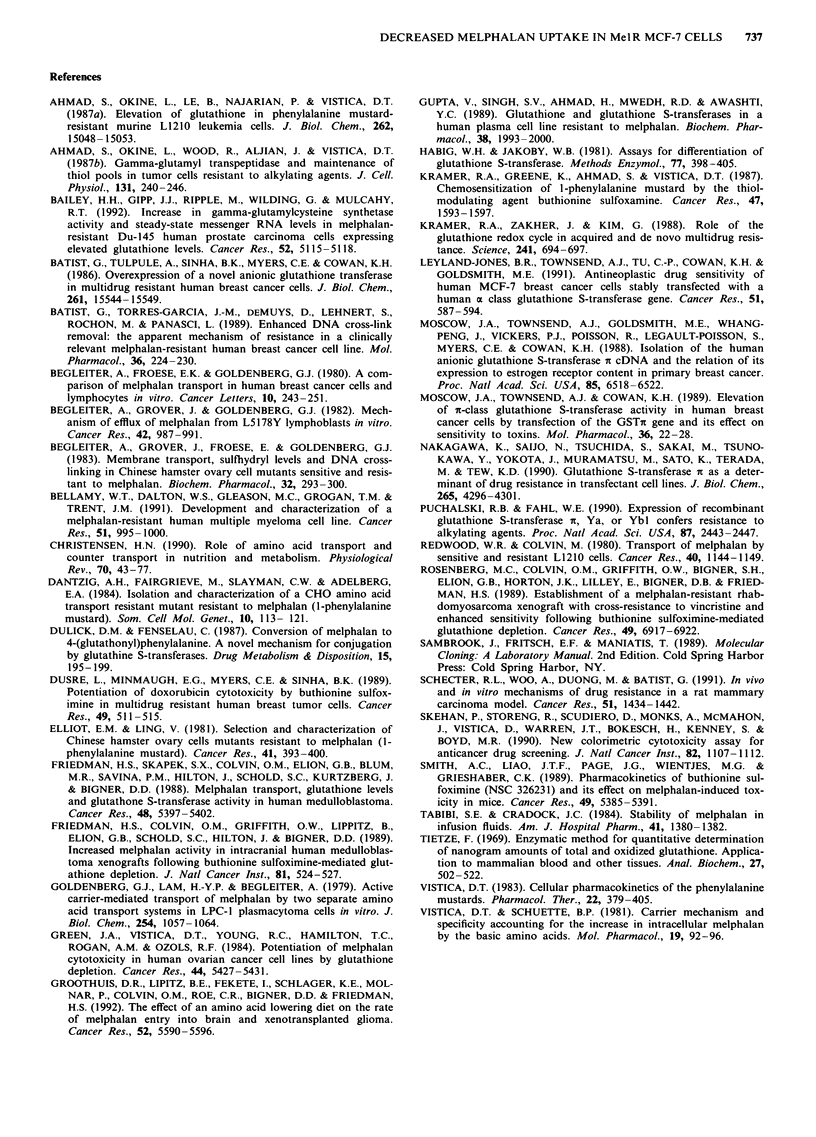# Decreased melphalan accumulation in a human breast cancer cell line selected for resistance to melphalan.

**DOI:** 10.1038/bjc.1993.419

**Published:** 1993-10

**Authors:** J. A. Moscow, C. A. Swanson, K. H. Cowan

**Affiliations:** Medical Breast Cancer Section, National Cancer Institute.

## Abstract

**Images:**


*Br. J. Cancer* (1993), **68**, 732–737 © Macmillan Press Ltd., 1993

### Decreased melphalan accumulation in a human breast cancer cell line selected for resistance to melphalan

J.A. Moscow, C.A. Swanson & K.H. Cowan

*Medical Breast Cancer Section, Medicine Branch, National Cancer Institute, Bethesda, Maryland, USA.*

**Summary** An *in vitro* model of acquired melphalan resistance was developed by serial incubation of an MCF-7 human breast cancer cell line in increasing concentrations of melphalan. The resulting derivative cell line, MelR MCF-7, was 30-fold resistant to melphalan. Uptake studies demonstrated decreased initial melphalan accumulation in MelR MCF-7 cells. Inverse-reciprocal plots of initial melphalan uptake revealed a 4-fold decrease in the apparent Vmax of MelR MCF-7 compared with WT MCF-7 (516 amol cell$^{-1}$ min$^{-1}$ *vs* 2110 amol cell$^{-1}$ min$^{-1}$ respectively) as well as a decrease in the apparent Kt (36 μM *vs* 70 μM respectively). Two amino acid transporters have previously been identified as melphalan transporters: system L, which is sodium-independent and inhibited by 2-amino-bicyclo[2,2,1]heptane-2-carboxylic acid (BCH), and system ASC which is sodium dependent and unaffected by BCH. At low concentrations of melphalan (3–30 μM), 1mM BCH competition eliminated the differences between the two cell lines, thus implicating an alteration of the system L transporter in the transport defect in the resistant cells. MelR MCF-7 cells were also evaluated for glutathione-mediated detoxification mechanisms associated with melphalan resistance. There was no difference between MelR MCF-7 and WT MCF-7 in glutathione content, glutathione-S-transferase activity and expression of pi class glutathione S-transferase RNA. In addition, buthionine sulfoximine did not reverse melphalan resistance in MelR MCF-7 cells. Therefore, MelR MCF-7 cells provide an *in vitro* model of transport-mediated melphalan resistance in human breast cancer cells.

Alkylating agent therapy is central to the chemotherapeutic approach to most malignancies, yet relatively few mechanisms of alkylating agent resistance have been described. In particular, while transport-mediated resistance has been well-characterised for many antineoplastic agents, most notably the multidrug resistance phenotype associated with the drug efflux pump P-glycoprotein, little is known about mechanisms of uptake, accumulation and efflux of alkylating agents. Cellular resistance to alkylating agents has generally been attributed to mechanisms which either detoxify the agent or repair its damage.

Melphalan (l-phenylalanine mustard, L-PAM, Alkeran) is a rationally designed alkylating agent which incorporates the amino acid phenylalanine as a part of its structure. Melphalan is active against ovarian cancer, myeloma, breast cancer and rhabdomyosarcoma. Most *in vitro* models of melphalan resistance have involved glutathione-mediated pathways, a finding observed in a wide variety of rodent cell lines including Chinese hamster ovary (Begleiter *et al.*, 1983) and murine L1210 leukaemia cells (Ahmad *et al.*, 1987*a*; Ahmad *et al.*, 1987*b*); and human cell lines, including ovarian (Green *et al.*, 1984), myeloma (Gupta *et al.*, 1989; Bellamy *et al.*, 1991) and prostate (Bailey *et al.*, 1992) cells.

In this report we characterise a melphalan resistant MCF-7 human breast cancer cell line (MelR MCF-7) which was isolated by serial incubation of the parental cell line in increasing concentrations of melphalan. This model of melphalan resistance differs from other human *in vitro* models of melphalan resistance in that the MelR MCF-7 cells have a significant defect in melphalan uptake associated with their resistance. In addition, unlike most other melphalan resistant cell lines reported, MelR MCF-7 cells have not developed changes in glutathione and glutathione-dependent pathways.

### Materials and methods

### *Cell and culture conditions*

WT MCF-7 and MelR MCF-7 cells were grown in Improved Minimal Essential Medium (IMEM) with (Gibco) and 5% (vol/vol) foetal calf serum (Gibco) as previously described (Batist *et al.*, 1986). Cells were maintained at 37°C in a 5% $CO_2$-95% air atmosphere.

Correspondence: J.A. Moscow, Building 10, Room 12N226, National Institutes of Health, Bethesda, MD 20892, USA.
Received 20 March 1993; and in revised form 9 June 1993.

### *Drugs*

Melphalan was obtained either from Burroughs Wellcome (Research Triangle Park, NC) or from Sigma, and was freshly prepared by dissolving aliquots in acidified ethanol at a concentration of 10 mg per 100 μl ethanol. BSO was obtained from the Drug Development Branch, NCI and Sigma.

### *Selection of melphalan-resistant MCF-7 cells*

MelR MCF-7 cells were isolated by serial incubation of WT MCF-7 cells in increasing concentrations of melphalan over a 14 month period. WT MCF-7 cells were plated and exposed to drug simultaneously. When surviving cells reached confluence, the cells were split and exposed to gradually increasing concentrations of drug. The starting melphalan concentration was 0.05 μM. At concentrations of 2 μM and 6 μM melphalan, the cells required repeated rescue with drug-free medium after plating the cells in drug. After several months a subpopulation emerged that could grow to confluence from a low cell density in medium containing 6 μM melphalan. The cells were then passaged in medium in which the melphalan concentration was gradually increased to 40 μM. MelR MCF-7 cells could not survive passages at concentrations greater than 60 μM despite several attempts. MelR MCF-7 cells were grown in drug-free medium for at least 1 week and as long as 2 months prior to cytotoxicity and drug accumulation studies.

### *Cytotoxicity and growth assays*

A semi-automated sulforhodamine dye-based microtiter-plate assay was used for cytotoxicity and growth assays. WT MCF-7 (3,000 cells/well) and MelR MCF-7 (6,000 cells/well) were plated into 96-well microtiter plates in 100 μl of IMEM with 5% foetal calf serum. On the following day, serial dilutions of melphalan were added in another 100 μl medium. The duration of exposure to melphalan was limited by the relatively brief half-life of the drug; in infusion fluids, the $t_{1/2}$ of melphalan at 37°C is approximately 3 h (Tabibi & Cradock, 1984). On the fifth day, the cells were fixed with 50 μl of 50% tricarboxylic acid for 1 h at 4°C, washed with water and allowed to air dry, stained with 0.4% sulforhodamine in 1% acetic acid for 10 min, washed five times with 1% acetic acid and allowed to dry (Skehan *et al.*, 1990). The stained cells were solubilised in 10 mM Tris base pH 10.5, and the absorbance at 540 nm was determined on a microplate reader (Skehan *et al.*, 1990). The survival fraction at a particular drug concentration was calculated as the percent of mean absorbance values relative to the mean absorbance values of cells grown in the absence of drug. The $IC_{50}$ value was calculated from the dose response curves as the concentration of drug which would produce a 50% decrease in the mean absorbance compared to the untreated wells. The relative resistance of MelR MCF-7 cells was expressed as the ratio of MelR MCF-7 $IC_{50}$ values to WT MCF-7 $IC_{50}$ values. All cytotoxicity assays were performed at least three separate times in triplicate. Cytotoxicity assays involving BSO were performed by adding 100 $\mu$M BSO to cells 4 h after plating and 24 h prior to the addition of melphalan. This exposure to BSO is comparable to that reported to significantly decrease glutathione levels in multidrug resistant MCF-7 cells (Kramer *et al.*, 1988; Dusre *et al.*, 1989).

Growth assays were also performed with the sulforhodamine technique. Cells were plated in medium in 96 well microtiter plates, and stained and fixed every 24 h. Doubling times were derived from the slopes of the linear part of each of the growth curves.

*Transport studies*

Melphalan uptake studies were performed as follows: WT MCF-7 and MelR MCF-7 cells were plated in either 6- or 12-well Linbro dishes. Approximately 48 h after plating, during the exponential growth phase, the cells were washed three times with PAG transport medium (Dulbecco's phosphate buffered saline containing 6.8 g $l^{-1}$ albumin and 1 g $l^{-1}$ glucose) pre-warmed to 37°C. Transport medium containing [$^{14}$C] melphalan (Moravek) was then added to the cells and incubated at 37°C for the specified time. At the end of the uptake period, the medium was quickly aspirated, and the plates were immersed in four consecutive baths of ice-cold Dulbecco's phosphate buffered saline in rapid succession. The plates were blotted dry, and the cells were solubilised by overnight incubation in 0.2 N NaOH at room temperature. The cell lysates were neutralised with 0.2 N HCl and the radioactivity determined by liquid scintillation counting. Amino acids used for transport inhibition studies were obtained from Gibco and BCH was obtained from Calbiochem. Competitors were added to the transport medium containing radiolabelled melphalan, so that cells were simultaneously exposed to radiolabelled melphalan and excess unlabelled competitor. Melphalan uptake at 0°C was minimal (less than 5% of the uptake at 37°C; Figure 2). The uptake at 0°C was determined and subtracted from the uptake measured at 37°C for each Lineweaver-Burke plot data point.

For uptake studies, the total number of cells was determined in replicate plates. Cells were trypsinised, resuspended in medium, passed several times through a 19 gauge needle to make a single cell suspension, diluted in isotonic buffered saline, and counted in a Coulter counter.

*Protein studies*

Cytosolic glutathione S-transferase activity was determined by using 1-chloro-2,4 dinitrobenzene as substrate (Habig & Jakoby, 1981). One unit of glutathione S-transferase enzyme activity is defined as the amount catalysing the conjugation of the substrate with glutathione at the rate of 1 nmol $min^{-1}$. Total glutathione levels were determined on cell cytosol by the cyclic reduction of oxidised glutathione with glutathione reductase and NADPH as described by Tietze (1969).

Cytosolic protein was extracted from washed cells by sonication and centrifugation of the cell pellet, and the protein concentration of the cytosols was determined spectrophotometrically using Coomassie Plus protein assay reagent (Pierce).

*Nucleic acid studies*

For Northern analysis, RNA was isolated by guanidine isothiocyanate-cesium chloride gradient centrifugation (Sambrook *et al.*, 1989) and the concentration was determined by spectrophotometry. The RNA samples (10 $\mu$g) were size fractionated on a 1% agarose gel that contained 2% formaldehyde using a buffer consisting of 20 mM MOPS containing 1 mM EDTA and 5 mM sodium acetate. Equivalence of RNA loading of the samples was confirmed by ethidium bromide staining of the gel. The RNA was transferred onto a Nytran membrane (Schleicher & Schuell), baked 2 h in an 80°C vacuum oven and hydridised overnight with a [$^{32}$P]-labelled cDNA probe for GSTP-1 (GST$\pi$-1; Moscow *et al.*, 1988). The blot was washed with a final stringency of 0.1 × SSC and 0.1% SDS at 65°C and hybridisation was detected by autoradiography.

### Results

*Selection of melphalan resistant MCF-7 cells*

Melphalan resistant MCF-7 cells were developed by serial incubation of MCF-7 cells in increasing concentrations of melphalan as described in Methods. The melphalan dose-response curve of the resulting subline, MelR MCF-7, is shown in Figure 1. The melphalan $IC_{50}$ of MelR MCF-7 cells is 52 $\mu$M, compared to 1.7 $\mu$M for WT MCF-7 cells. Thus, MelR MCF-7 cells are 30-fold resistant to melphalan at the $IC_{50}$ level.

MelR MCF-7 cells have a lower plating efficiency than WT MCF-7 cells, 31 ± 1% and 51 ± 7% respectively. In addition, MelR MCF-7 cells grow slower than WT MCF-7 cells, with a doubling time of 47 ± 3 h *vs* 27 ± 1 h for MelR MCF-7 and WT MCF-7 cells respectively. MelR MCF-7 cells contain slightly more cytosolic protein per cell than the parental cell line (101 ± 7 *vs* 80 ± 1 $\mu$g $10^{-6}$ cells).

Resistance to melphalan in MelR MCF-7 cells gradually declined when the cells were maintained in the absence of drug. After 2 months passage in the absence of drug, resistance to melphalan decreased to an $IC_{50}$ of 40 $\mu$M and further decreased to an $IC_{50}$ of 20 $\mu$M after 4 months passage without exposure to the selecting agent. Therefore, after 4 months passage out of drug, MelR MCF-7 cells retained 12-fold level of resistance to melphalan in comparison to WT MCF-7 cells. The gradual loss of resistance seen in MelR MCF-7 cells when passaged out of drug suggests that resistance may be the result of gene amplification. Cytogenetic analysis of MelR MCF-7 cells has revealed minute chromosomes in 17 of 30 metaphases examined (W. Peterson, personal communication).

*Melphalan transport studies*

The cellular uptake of 50 $\mu$M melphalan over a 30 min time course is shown in Figure 2. This plot demonstrates a 4-fold decrease in melphalan accumulation in MelR MCF-7 cells in

**Figure 1** Cytotoxicity assay of melphalan on WT MCF-7 and MelR MCF-7 cells. Cell growth after continuous exposure to melphalan was determined relative to untreated controls using a sulforhodamine dye assay as described in the Methods section. The graph indicates the mean ± s.d. of six separate determinations performed in triplicate.

comparison to WT MCF-7 cells. The uptake appears to be linear over the first 6 min, and then reaches a plateau by 20 min. The time over which linear uptake occurs is longer than that observed in L1210 cells (Redwood & Colvin, 1980), but comparable to that previously observed in MCF-7 cells (Begleiter *et al.*, 1980). Melphalan uptake at 4°C was minimal (Figure 2).

An inverse-reciprocal plot of melphalan uptake at 2 min over a concentration range of 1–300 μM is shown in Figure 3. The Kt and Vmax for melphalan for each cell line was determined by linear regression analysis. For WT MCF-7 cells, the apparent Kt was 70 μM and the Vmax was 2110 amol $cell^{-1}$ $min^{-1}$. For MelR MCF-7 cells, the Kt was 36 μM and the Vmax was 516 amol $cell^{-1}$ $min^{-1}$. Therefore, while there may be some qualitative changes in the apparent Kt, the major difference between MelR MCF-7 and WT MCF-7 cell lines appears to be related to the 4-fold decrease in the Vmax.

Previous studies of melphalan uptake have attributed melphalan uptake to two amino acid transport systems (Goldenberg *et al.*, 1979; reviewed by Vistica, 1983). One transporter is similar to the amino acid transport System L which preferentially transports leucine, but also transports phenylalanine, tyrosine, tryptophan and valine. System L is inhibited by the synthetic inert amino acid BCH and is sodium independent. The other transporter is similar, if not identical, to System ASC (for alanine, serine, cysteine) which is sodium dependent and unaffected by BCH.

In order to determine which amino acid transport system was responsible for melphalan uptake in WT MCF-7 and MelR MCF-7 cells, we examined initial uptake in the absence of sodium and in the presence of BCH. Initial melphalan (100 μM) uptake when choline was substituted for sodium in the transport medium was 102 ± 1% in WT MCF-7 cells, and 81 ± 9% in MelR MCF-7 cells relative to uptake of drug measured in PAG transport medium. Thus, a sodium-independent mechanism accounts for most, if not all, of the melphalan transport in both WT MCF-7 and MelR MCF-7 cells.

The effect of BCH inhibition of melphalan uptake is shown in Figure 4. As can be seen by the inverse reciprocal plots, 1 mM BCH eliminates the difference between MelR MCF-7 and WT MCF-7 cells in the initial melphalan uptake over the concentration range of 3 to 30 μM. This finding suggests that the difference in melphalan uptake between the two cell lines can be ascribed to an alteration in the System L transporter. BCH competition studies, seen in both Figures 4 and 5, also demonstrate that non-System L-mediated transport is a small but significant mechanism of melphalan uptake in both cell lines.

Melphalan uptake competition studies in the presence of excess unlabelled amino acids (Figure 5) supports the importance of the System L transporter in the two cell lines. System L substrates, such as leucine, phenylalanine, tyrosine and tryptophan, were more effective in inhibiting initial melphalan uptake than the amino acids which are poor sub-

**Figure 2** Melphalan accumulation in WT MCF-7 and MelR MCF-7 cells. Uptake of 50 μM melphalan was determined from 0.5 to 30 min in PAG transport medium at 37°C and 4°C as described in the Methods section. The graph indicates the mean ± s.d. of two separate determinations performed in duplicate.

**Figure 3** An inverse-reciprocal plot of melphalan uptake between 1–300 μM in WT MCF-7 and MelR MCF-7 cells. Initial melphalan uptake was measured at 2 min at 37°C as described in the Methods section. The linear regression solutions are for WCT MCF-7 y = 4.714e$^{-4}$ + 3.3187e$^{-2}$x with a regression coefficient of 0.999; and for MelR MCF-7 y = 1.9386e$^{-3}$ + 70449e$^{-2}$x with a regression coefficient of 0.999. The graph indicates the mean ± s.d. of four separate determinations performed in triplicate.

**Figure 4** An inverse reciprocal plot of initial 2 min uptake of 3 to 30 μM melphalan in MelR MCF-7 and WT MCF-7 cells in the presence and in the absence of 1 mM BCH. The linear regression solutions are: WT MCF-7, y = 1.5943e$^{-4}$ + 3.6310e$^{-2}$x with a regression coefficient of 0.996; WT MCF-7 in 1 mM BCH, y = 4.4915e$^{-3}$ + 0.1556x with a regression coefficient of 0.990; MelR MCF-7, y = 1.4266e$^{-3}$ + 7.857e$^{-2}$x with a regression coefficient of 1.000; and MelR MCF-7 in 1 mM BCH, y = 3.6165e$^{-3}$ + 0.1511x with a regression coefficient of 0.994.

**Figure 5** Bar graph representation of inhibition of initial 2 min uptake of 100 μM melphalan by 300 μM of various competitors at 37°C. MEl, melphalan; cys, cystine; ser, serine; arg, arginine; glu, glutamine; his, histidine; val, valine; try, tryptophan; tyr, tyrosine; leu, leucine; val, valine. The graph indicates the mean ± s.d. of at least three separate determinations performed in duplicate or triplicate.

strates for System L, such as arginine, cystine and serine (Christensen, 1990).

Melphalan efflux was examined in both cell lines after incubation in radiolabelled melphalan. As shown in Figure 6, there was no difference in melphalan efflux between the two cell lines after the initial loading period. Therefore, drug efflux does not appear to contribute to the decreased melphalan accumulation seen in MelR MCF-7 cells.

### *Glutathione-dependent detoxification*

We examined MelR MCF-7 cells for alterations in glutathione and its dependent enzymes. As shown in Table I, there was no significant difference between the two cell lines in either glutathione content or glutathione S-transferase activity. A Northern analysis of the expression of GSTP1-1 RNA is shown in Figure 7. There was no detectable expression of GSTP1-1 RNA in either cell line.

Most, if not all, melphalan-resistant cell lines with alterations in glutathione-dependent pathways demonstrate reversal of resistance with BSO, a glutathione synthesis inhibitor. The effect of preincubation of WT MCF-7 and MelR MCF-7 cells with BSO on melphalan cytotoxicity is shown in Figure 8. BSO did not specifically reverse the melphalan resistance of MelR MCF-7 cells, indicating that melphalan resistance in MelR MCF-7 cells is not mediated by glutathione-dependent pathways.

### Discussion

We have isolated a melphalan resistant MCF-7 human breast cancer cell line by serial incubation of MCF-7 cells in increasing concentrations of melphalan. The resulting cell line, MelR MCF-7, is 30-fold resistant to melphalan. Characterisation of this cell line has revealed that resistance is associated with a decrease in melphalan accumulation resulting from diminished accumulation of drug, and that glutathione-dependent mechanisms apparently are not responsible for the acquired resistance seen in MelR MCF-7 cells. It is possible that other unidentified mechanisms of melphalan resistance co-exist with decreased melphalan transport in MelR MCF-7 cells.

A study by Begleiter *et al.* (1980) has previously examined melphalan uptake in WT MCF-7 cells. The time course of initial melphalan uptake was very similar to the one presented in this study, with linear uptake for approximately the first 5 min. The Kt values were similar, 54 μM (BCH sensitive) *vs* 70 μM reported here. The Vmax is different in the two

**Figure 6** Efflux of melphalan from WT MCF-7 and MelR MCF-7 cells. Cells were incubated in duplicate or triplicate wells in 100 μM melphalan in PAG transport medium at 37°C for 30 min. The medium was changed to PAG medium without drug and replicate plates were examined over the time course for melphalan retention. Values are expressed as a percent of retained melphalan relative to the intracellular melphalan present at the end of the loading period. The graph indicates the mean ± s.d. of two separate determinations performed in triplicate.

**Table I** Glutathione and glutathione S-transferase in MelR MCF-7 cells

| | *GSH content nmoles mg$^{-1}$ protein* | *GST activity units mg$^{-1}$ protein* |
|---|---|---|
| WT MCF-7 | 52 ± 7 | 7.4 ± 1.4 |
| MelR MCF-7 | 43 ± 12 | 3.7 ± 0.8 |

**Figure 7** Northern analysis of GSTP1-1 (GSTπ) RNA expression in MelR MCF-7 cells. Ten μg of RNA was probed for expression of GSTP1-1 RNA as described in Materials and methods. RNA from the multidrug resistant MCF-7 subline AdrR MCF-7, which overexpresses GSTP1-1 (Batist, 1986) was used for a positive control.

**Figure 8** Cytotoxicity assay of melphalan on WT MCF-7 and MelR MCF-7 cells in the presence and in the absence of BSO. Cells in triplicate wells were incubated with BSO 100 μM for 24 h prior to exposure to melphalan. The graph indicates the mean ± s.d. of five separate determinations performed in triplicate.

reports, 700 amol cell$^{-1}$ min$^{-1}$ *vs* 2110 amol cell$^{-1}$ min$^{-1}$ in this study. In both studies, there is evidence that melphalan uptake is mediated by at least two different transport systems, one which is BCH-sensitive and which accounts for most melphalan uptake at low ($\leqslant 30\ \mu$M) melphalan concentrations, and a BCH-insensitive system. Although the BCH-insensitive system resembled system ASC in the report by Begleiter *et al.* (1980), in that melphalan uptake in their MCF-7 cell line was both partially sodium-dependent and inhibited by glutamine excess, in our study we found no evidence of sodium-dependent melphalan uptake in WT MCF-7 cells.

Several studies have previously demonstrated an association between altered system L transport and melphalan resistance. Redwood and Colvin (1980) reported an *in vivo* model of melphalan resistance in a murine L1210 leukaemia cell line selected for melphalan resistance while grown intraperitoneal in mice. Strikingly, the L1210 cell lines displayed a response to BCH inhibition of system L virtually identical to that seen in MelR MCF-7 cells. These parallel observations are even more remarkable considering the fact that the Vmax in WT MCF-7 cells is 10- to 80-fold higher than the Vmax reported for L1210 cells.

Using an alternative approach, Dantzig *et al.* (1984) isolated a Chinese hamster ovary cell line with defective system L transport by selecting cells with slow growth characteristics after treatment with a mutagen and exposure to medium containing relatively low concentrations of leucine. A single isolated clone demonstrated decreased uptake of system L substrates and relative melphalan resistance under drug exposure conditions designed to limit non-system L melphalan uptake.

Two human medulloblastoma cell lines with differences in relative sensitivity to melphalan have been compared to each with respect to melphalan transport and glutathione-related characteristics (Friedman *et al.*, 1988). The comparison of these cell lines indicated an association between melphalan resistance and a decreased Vmax for melphalan, although both system L and system ASC were functional in these cell lines.

Enhanced melphalan efflux has also been associated with melphalan resistance. Analysis of a Chinese hamster ovary cell line selected for colchicine resistance and found to be cross-resistant to melphalan (Elliot & Ling, 1981) revealed that decreased melphalan accumulation resulted from enhanced melphalan efflux (Begleiter *et al.*, 1983). However, analysis of melphalan efflux from WT MCF-7 and MelR MCF-7 cells in the presence of PAG transport medium (Figure 6) revealed no differences between the WT MCF-7 cells and the resistant subline. Melphalan efflux is a complicated process which can be affected by the concentrations of extracellular amino acids (Begleiter *et al.*, 1982; Vistica & Schuette, 1981). However, the sensitive and resistant cell lines did not differ in the rate of efflux when incubated in amino acid replete IMEM growth medium after initial melphalan loading (data not shown).

MelR MCF-7 cells therefore represent the first *in vitro* model of transport-associated melphalan resistance in a human cell line selected for resistance to melphalan. This cell line also demonstrates that altered system L-mediated transport may be a relevant mechanism of acquired resistance to melphalan in human tumours. In contrast to system ASC, system L-mediated transport appears to be responsible for acquired resistance in every model of melphalan resistance in which melphalan uptake is impaired. Therefore, augmentation of system L capacity may be an appropriate strategy for circumventing melpahalan resistance or increasing melphalan cytotoxicity.

Glutathione and glutathione-dependent enzymes have frequently been associated with melphalan resistance. Increased glutathione levels have been observed in a wide variety of cell lines selected for melphalan resistance (Ahmad *et al.*, 1987*a*; Bailey *et al.*, 1992; Bellamy *et al.*, 1991; Green *et al.*, 1984; Rosenberg *et al.*, 1989; Schecter *et al.*, 1991). Two other models of melphalan resistance have been reported in which no increase in glutathione content was found in the resistant cell lines (Friedman *et al.*, 1988; Gupta *et al.*, 1989). In cell lines that demonstrate an increase in glutathione levels, BSO has been found to consistently reverse melphalan resistance (Ahmad *et al.*, 1987*a*; Bellamy *et al.*, 1991; Green *et al.*, 1984; Rosenberg *et al.*, 1989).

The involvement of GSTs in melphalan resistance was suggested by biochemical studies which demonstrated that GSTs could conjugate melphalan to 4-(glutathionyl)phenylalanine (Dulick & Fenselau, 1987). The association between GSTs and models of melphalan resistance has been inconsistent, with an increase in GST activity reported in two cell lines (Gupta *et al.*, 1989; Schecter *et al.*, 1991) but not in others (Friedman *et al.*, 1988; Rosenberg *et al.*, 1989). In the models of melphalan resistance in which GST activity was elevated, the increase has been associated with an increase in the pi-class (Gupta *et al.*, 1989; Schecter *et al.*, 1991) and alpha class (Schecter *et al.*, 1991) GST isozymes. However, the greatest level of melphalan resistance conferred by transfection of pi and alpha class GST genes was 1.5-fold (Puchalski & Fahl, 1990), while other studies showed no acquisition of melphalan resistance in GST transfected clones (Leyland-Jones *et al.*, 1991; Moscow *et al.*, 1989; Nakagawa *et al.*, 1990). Two other glutathione dependent enzymes have also been associated with melphalan resistance, gamma glutamyl transpeptidase (Ahmad *et al.*, 1987*b*) and gamma glutamyl cysteine synthetase (Bailey *et al.*, 1992).

Clinical trials combining BSO and melphalan are currently underway. The use of BSO to decrease glutathione levels and enhance its antineoplastic cytotoxicity has been successful not only *in vitro*, but also in animal models (Friedman *et al.*, 1989; Kramer *et al.*, 1987). Unfortunately, normal cells may also employ glutathione-mediated defences, and BSO can add to melphalan mediated host toxicity (Smith *et al.*, 1989). BSO may not be effective in clinical trials if it does not increase the therapeutic index of melphalan, or alternatively, if malignant tumours develop mechanisms of resistance to melphalan that are not glutathione-dependent. For example, neither of the *in vitro* models of melphalan resistance in MCF-7 human breast cancer cells, the study presented here and a 3-fold resistant subline reported by Batist *et al.* (1989), appear to utilise glutathione-related defences. In contrast to MelR MCF-7 cells, the melphalan resistant cell line reported by Batist *et al.* does not demonstrate a change in melphalan uptake; resistance was attributed to an apparent change in DNA repair capacity.

In summary, MelR MCF-7 cells represent a useful *in vitro* model of melphalan resistance mediated by decreased capacity of the system L amino acid transporter. Melphalan is administered in a mileau of competitive inhibitors of its uptake. The potential utility of manipulation of amino acid transport systems in conjunction with melphalan chemotherapy was recently illustrated by a study which demonstrated increased melphalan uptake in tumour xenografts after circulating amino acid levels were lowered through fasting and administration of a protein-free diet (Groothuis *et al.*, 1992). Such strategies may ultimately improve the therapeutic effectiveness of melphalan. MelR MCF-7 cells provide an *in vitro* model for developing methods of specifically increasing melphalan uptake by modulating system L activity.

We wish to thank Drs Edward Minmaugh and Alan Townsend for their assistance in performing glutathione and GST assays.

**References**

AHMAD, S., OKINE, L., LE, B., NAJARIAN, P. & VISTICA, D.T. (1987*a*). Elevation of glutathione in phenylalanine mustard-resistant murine L1210 leukemia cells. *J. Biol. Chem.*, **262**, 15048–15053.

AHMAD, S., OKINE, L., WOOD, R., ALJIAN, J. & VISTICA, D.T. (1987*b*). Gamma-glutamyl transpeptidase and maintenance of thiol pools in tumor cells resistant to alkylating agents. *J. Cell. Physiol.*, **131**, 240–246.

BAILEY, H.H., GIPP, J.J., RIPPLE, M., WILDING, G. & MULCAHY, R.T. (1992). Increase in gamma-glutamylcysteine synthetase activity and steady-state messenger RNA levels in melphalan-resistant Du-145 human prostate carcinoma cells expressing elevated glutathione levels. *Cancer Res.*, **52**, 5115–5118.

BATIST, G., TULPULE, A., SINHA, B.K., MYERS, C.E. & COWAN, K.H. (1986). Overexpression of a novel anionic glutathione transferase in multidrug resistant human breast cancer cells. *J. Biol. Chem.*, **261**, 15544–15549.

BATIST, G., TORRES-GARCIA, J.-M., DEMUYS, D., LEHNERT, S., ROCHON, M. & PANASCI, L. (1989). Enhanced DNA cross-link removal: the apparent mechanism of resistance in a clinically relevant melphalan-resistant human breast cancer cell line. *Mol. Pharmacol.*, **36**, 224–230.

BEGLEITER, A., FROESE, E.K. & GOLDENBERG, G.J. (1980). A comparison of melphalan transport in human breast cancer cells and lymphocytes *in vitro*. *Cancer Letters*, **10**, 243–251.

BEGLEITER, A., GROVER, J. & GOLDENBERG, G.J. (1982). Mechanism of efflux of melphalan from L5178Y lymphoblasts *in vitro*. *Cancer Res.*, **42**, 987–991.

BEGLEITER, A., GROVER, J., FROESE, E. & GOLDENBERG, G.J. (1983). Membrane transport, sulfhydryl levels and DNA cross-linking in Chinese hamster ovary cell mutants sensitive and resistant to melphalan. *Biochem. Pharmacol.*, **32**, 293–300.

BELLAMY, W.T., DALTON, W.S., GLEASON, M.C., GROGAN, T.M. & TRENT, J.M. (1991). Development and characterization of a melphalan-resistant human multiple myeloma cell line. *Cancer Res.*, **51**, 995–1000.

CHRISTENSEN, H.N. (1990). Role of amino acid transport and counter transport in nutrition and metabolism. *Physiological Rev.*, **70**, 43–77.

DANTZIG, A.H., FAIRGRIEVE, M., SLAYMAN, C.W. & ADELBERG, E.A. (1984). Isolation and characterization of a CHO amino acid transport resistant mutant resistant to melphalan (1-phenylalanine mustard). *Som. Cell Mol. Genet.*, **10**, 113– 121.

DULICK, D.M. & FENSELAU, C. (1987). Conversion of melphalan to 4-(glutathonyl)phenylalanine. A novel mechanism for conjugation by glutathine S-transferases. *Drug Metabolism & Disposition*, **15**, 195–199.

DUSRE, L., MINMAUGH, E.G., MYERS, C.E. & SINHA, B.K. (1989). Potentiation of doxorubicin cytotoxicity by buthionine sulfoximine in multidrug resistant human breast tumor cells. *Cancer Res.*, **49**, 511–515.

ELLIOT, E.M. & LING, V. (1981). Selection and characterization of Chinese hamster ovary cells mutants resistant to melphalan (1-phenylalanine mustard). *Cancer Res.*, **41**, 393–400.

FRIEDMAN, H.S., SKAPEK, S.X., COLVIN, O.M., ELION, G.B., BLUM, M.R., SAVINA, P.M., HILTON, J., SCHOLD, S.C., KURTZBERG, J. & BIGNER, D.D. (1988). Melphalan transport, glutathione levels and glutathone S-transferase activity in human medulloblastoma. *Cancer Res.*, **48**, 5397–5402.

FRIEDMAN, H.S., COLVIN, O.M., GRIFFITH, O.W., LIPPITZ, B., ELION, G.B., SCHOLD, S.C., HILTON, J. & BIGNER, D.D. (1989). Increased melphalan activity in intracranial human medulloblastoma xenografts following buthionine sulfoximine-mediated glutathione depletion. *J. Natl Cancer Inst.*, **81**, 524–527.

GOLDENBERG, G.J., LAM, H.-Y.P. & BEGLEITER, A. (1979). Active carrier-mediated transport of melphalan by two separate amino acid transport systems in LPC-1 plasmacytoma cells *in vitro*. *J. Biol. Chem.*, **254**, 1057–1064.

GREEN, J.A., VISTICA, D.T., YOUNG, R.C., HAMILTON, T.C., ROGAN, A.M. & OZOLS, R.F. (1984). Potentiation of melphalan cytotoxicity in human ovarian cancer cell lines by glutathione depletion. *Cancer Res.*, **44**, 5427–5431.

GROOTHUIS, D.R., LIPITZ, B.E., FEKETE, I., SCHLAGER, K.E., MOLNAR, P., COLVIN, O.M., ROE, C.R., BIGNER, D.D. & FRIEDMAN, H.S. (1992). The effect of an amino acid lowering diet on the rate of melphalan entry into brain and xenotransplanted glioma. *Cancer Res.*, **52**, 5590–5596.

GUPTA, V., SINGH, S.V., AHMAD, H., MWEDH, R.D. & AWASHTI, Y.C. (1989). Glutathione and glutathione S-transferases in a human plasma cell line resistant to melphalan. *Biochem. Pharmacol.*, **38**, 1993–2000.

HABIG, W.H. & JAKOBY, W.B. (1981). Assays for differentiation of glutathione S-transferase. *Methods Enzymol.*, **77**, 398–405.

KRAMER, R.A., GREENE, K., AHMAD, S. & VISTICA, D.T. (1987). Chemosensitization of 1-phenylalanine mustard by the thiol-modulating agent buthionine sulfoxamine. *Cancer Res.*, **47**, 1593–1597.

KRAMER, R.A., ZAKHER, J. & KIM, G. (1988). Role of the glutathione redox cycle in acquired and de novo multidrug resistance. *Science*, **241**, 694–697.

LEYLAND-JONES, B.R., TOWNSEND, A.J., TU, C.-P., COWAN, K.H. & GOLDSMITH, M.E. (1991). Antineoplastic drug sensitivity of human MCF-7 breast cancer cells stably transfected with a human α class glutathione S-transferase gene. *Cancer Res.*, **51**, 587–594.

MOSCOW, J.A., TOWNSEND, A.J., GOLDSMITH, M.E., WHANG-PENG, J., VICKERS, P.J., POISSON, R., LEGAULT-POISSON, S., MYERS, C.E. & COWAN, K.H. (1988). Isolation of the human anionic glutathione S-transferase π cDNA and the relation of its expression to estrogen receptor content in primary breast cancer. *Proc. Natl Acad. Sci. USA*, **85**, 6518–6522.

MOSCOW, J.A., TOWNSEND, A.J. & COWAN, K.H. (1989). Elevation of π-class glutathione S-transferase activity in human breast cancer cells by transfection of the GSTπ gene and its effect on sensitivity to toxins. *Mol. Pharmacol.*, **36**, 22–28.

NAKAGAWA, K., SAIJO, N., TSUCHIDA, S., SAKAI, M., TSUNOKAWA, Y., YOKOTA, J., MURAMATSU, M., SATO, K., TERADA, M. & TEW, K.D. (1990). Glutathione S-transferase π as a determinant of drug resistance in transfectant cell lines. *J. Biol. Chem.*, **265**, 4296–4301.

PUCHALSKI, R.B. & FAHL, W.E. (1990). Expression of recombinant glutathione S-transferase π, Ya, or Yb1 confers resistance to alkylating agents. *Proc. Natl Acad. Sci. USA*, **87**, 2443–2447.

REDWOOD, W.R. & COLVIN, M. (1980). Transport of melphalan by sensitive and resistant L1210 cells. *Cancer Res.*, **40**, 1144–1149.

ROSENBERG, M.C., COLVIN, O.M., GRIFFITH, O.W., BIGNER, S.H., ELION, G.B., HORTON, J.K., LILLEY, E., BIGNER, D.B. & FRIEDMAN, H.S. (1989). Establishment of a melphalan-resistant rhabdomyosarcoma xenograft with cross-resistance to vincristine and enhanced sensitivity following buthionine sulfoximine-mediated glutathione depletion. *Cancer Res.*, **49**, 6917–6922.

SAMBROOK, J., FRITSCH, E.F. & MANIATIS, T. (1989). *Molecular Cloning: A Laboratory Manual*. 2nd Edition. Cold Spring Harbor Press: Cold Spring Harbor, NY.

SCHECTER, R.L., WOO, A., DUONG, M. & BATIST, G. (1991). *In vivo* and *in vitro* mechanisms of drug resistance in a rat mammary carcinoma model. *Cancer Res.*, **51**, 1434–1442.

SKEHAN, P., STORENG, R., SCUDIERO, D., MONKS, A., MCMAHON, J., VISTICA, D., WARREN, J.T., BOKESCH, H., KENNEY, S. & BOYD, M.R. (1990). New colorimetric cytotoxicity assay for anticancer drug screening. *J. Natl Cancer Inst.*, **82**, 1107–1112.

SMITH, A.C., LIAO, J.T.F., PAGE, J.G., WIENTJES, M.G. & GRIESHABER, C.K. (1989). Pharmacokinetics of buthionine sulfoximine (NSC 326231) and its effect on melphalan-induced toxicity in mice. *Cancer Res.*, **49**, 5385–5391.

TABIBI, S.E. & CRADOCK, J.C. (1984). Stability of melphalan in infusion fluids. *Am. J. Hospital Pharm.*, **41**, 1380–1382.

TIETZE, F. (1969). Enzymatic method for quantitative determination of nanogram amounts of total and oxidized glutathione. Application to mammalian blood and other tissues. *Anal. Biochem.*, **27**, 502–522.

VISTICA, D.T. (1983). Cellular pharmacokinetics of the phenylalanine mustards. *Pharmacol. Ther.*, **22**, 379–405.

VISTICA, D.T. & SCHUETTE, B.P. (1981). Carrier mechanism and specificity accounting for the increase in intracellular melphalan by the basic amino acids. *Mol. Pharmacol.*, **19**, 92–96.